# Genome-wide characterization of microsatellites in Triticeae species: abundance, distribution and evolution

**DOI:** 10.1038/srep32224

**Published:** 2016-08-26

**Authors:** Pingchuan Deng, Meng Wang, Kewei Feng, Licao Cui, Wei Tong, Weining Song, Xiaojun Nie

**Affiliations:** 1 State Key Laboratory of Crop Stress Biology in Arid Areas, College of Agronomy and Yangling Branch of China Wheat Improvement Center, Northwest A&F University, Yangling, Shaanxi 712100, China; 2 Australia-China Joint Research Centre for Abiotic and Biotic Stress Management in Agriculture, Horticulture and Forestry, Yangling, Shaanxi 712100, China

## Abstract

Microsatellites are an important constituent of plant genome and distributed across entire genome. In this study, genome-wide analysis of microsatellites in 8 Triticeae species and 9 model plants revealed that microsatellite characteristics were similar among the Triticeae species. Furthermore, genome-wide microsatellite markers were designed in wheat and then used to analyze the evolutionary relationship of wheat and other Triticeae species. Results displayed that *Aegilops tauschii* was found to be the closest species to *Triticum aestivum*, followed by *Triticum urartu*, *Triticum turgidum* and *Aegilops speltoides*, while *Triticum monococcum*, *Aegilops sharonensis* and *Hordeum vulgare* showed a relatively lower PCR amplification effectivity. Additionally, a significantly higher PCR amplification effectivity was found in chromosomes at the same subgenome than its homoeologous when these markers were subjected to search against different chromosomes in wheat. After a rigorous screening process, a total of 20,666 markers showed high amplification and polymorphic potential in wheat and its relatives, which were integrated with the public available wheat markers and then anchored to the genome of wheat (CS). This study not only provided the useful resource for SSR markers development in Triticeae species, but also shed light on the evolution of polyploid wheat from the perspective of microsatellites.

Microsatellites or tandem simple sequence repeats (SSRs), as iterations of 1–6 bp nucleotide motifs, were associated with replication slippage and DNA repair mechanisms and widely detected in the genomes of prokaryotic and eukaryotic organisms^1^,^2^. Microsatellites have been initially regarded as ‘junk DNA’ or mainly used as ‘neutral’ genetic marker^3^, while recent studies have documented that they could play crucial roles in affecting gene activity, chromatin organization and DNA metabolic processes^4^. Besides their direct biological functions, microsatellites have been proven to be a rich source of hypervariable codominant markers because they were subjected to a high rate of single-motif insertion and deletion mutations. For the past 15 years, microsatellite markers were extensively used in many research areas such as quantitative trait loci (QTL) mapping, genetic diversity studies, marker-assisted selection and evolutionary studies as their obvious advantages, such as high abundance, dispersion throughout the entire genome, codominant inheritance and reproducibility as well as specificity^5^,^6^.

The Triticeae tribe, belonging to Pooideae subfamily of Poaceae family, has the most significant economic and agricultural importance. With about five hundred wild and domesticated species, it not only included the three major cereals worldwide (wheat, barley and rye), but also included a number of forage and pasture grasses^7^. *Triticum*, an important constituent of Triticeae tribe, mainly included one diploid species, *T. urartu* (AA, n = 7) and three allopolyploid species, *T. dicoccoides* (AABB, n = 14), *T. turgidum* (AABB, n = 14) and *T. aestivum* (AABBDD, n = 21)^7^,^8^. Majority of the studies supported that *T. dicoccoides*, originated from the chromosome doubling after the natural hybridization between *T. urartu* and *A. speltoides*. Then, *T. dicoccoides* were domesticated to *T. turgidum*, which was further hybridized with *A. tauschii* and given rise to *T. aestivum*^9^,^10^,^11^. The combination of homoelogous chromosomes from divergent species not only promotes functional divergence of duplicate genes, but also generates heterozygosity and novel interactions leading to genetic and phenotypic variability^12^. There have been numerous studies devoted to understanding the mechanisms and evolution of polyploidy in *T. aestivum*^13^,^14^,^15^, while the roles of microsatellites have not been well understood.

Initially, SSR markers were developed from screening with SSR positive clones in genomic DNA library, but rather difficult and laborious. Then, bacterial artificial chromosome (BAC) end sequences^16^ and expressed sequence tags (ESTs)^5^,^9^,^17^,^18^ were widely used to SSR markers development in most plant species, which showed lower cost and effort but might yield multiple sets of markers at the same locus. Thanks to the rapid development of genome sequencing technology, the availability and analysis of nearly complete genome sequences from many organisms has provided insight into the distribution, putative function of microsatellites and also markers development^19^,^20^,^21^,^22^,^23^,^24^,^25^. More recently, the genome sequences were currently available for 8 Triticeae species, including *Triticum aestivum* and its seven relatives, which provided the opportunity to genome-wide characterized the distribution and frequency of microsatellites in Triticeae.

In present study, genome sequences of 8 Triticeae species and 9 model plants were mined for the abundance and composition of microsatellites. The nature of these microsatellites was analyzed and compared based on the genome sequences in the respective specie. Then, primers flanking these microsatellite motifs in *T. aestivum* were designed and used to analyze the relationship among wheat and its relatives. In order to improve the utilization efficiency of newly developed markers, only the longer perfect repeats (SSRs ≥ 20 nucleotides in length) and non-mononucleotides were selected to final genome-wide SSR markers development in wheat. *In silico* analysis of conservation and cross-transferability of these markers among barley and wheat close relatives were also carried out to study their utility in comparative mapping of genes and genomes. Our study not only provided the rich resource for SSR markers development in Triticeae species, but also provided the important information on the evolution of polyploid wheat.

## Results and Discussion

### Characteristics and frequency of microsatellites in Triticeae species

In current study, microsatellite distribution was characterized and compared in 18 genomes. A total of 4,763,266 microsatellites were identified, with an overall frequency of 126.83 per Mb (Table 1). The variation in the microsatellite frequencies of these Poaceae species was 3.77-fold, which was highly similar to that reported for angiosperm species (3.7-fold)^26^. Moreover, the Poaceae species with large genome sizes have a low or moderate microsatellite frequency, which was agreed well with the significantly negative correlation between microsatellite frequencies and genome sizes (r = −0.464)^26^. Interesting, we found that GC contents also showed a significantly negative correlations with microsatellite number or frequency (r = −0.510).

The microsatellite characteristics (e.g., frequency and distributions of microsatellites with respect to motif length, type and repeat number) were generally similar among the Poaceae species. Comparative analysis of the occurrences of various microsatellites revealed that 80% of Poaceae species were rich in mononucleotide repeats (MNRs), while wheat (*T. aestivum*) and its progenitors (*T. urartu* and *A. tauschii*) have higher frequencies of DNRs (Table 1). Conversely, Shi *et al*. (2013) reported that TNRs and TeNRs were the most abundant for Monocotyledoneae species, while MNRs and DNRs displayed relatively high proportions for Dicotyledoneae species^26^. The difference in the microsatellite characteristics could be due to the different criteria used to identify SSRs in the database mining. For example, 10 and 11 copies, accounting for 67.63% of the total number of mononucleotide, were remained in our study while removed by Shi *et al*. (2013).

The distributions with respect to the dominant/major motif type of microsatellites were showed in Figure S1. Specifically, the dominant motif type was rich in A/T, AG/CT, AAG/CTT, AAAT/ATTT, AAAAG/CTTTT and AGATAT/ATATCT. The distributions with respect to the dominant/major motif type of microsatellites were almost identical for MNRs, DNRs and PNRs, whereas TNRs, TeNRs and HNRs were relatively uncommon among Poaceae species. For example, AG/CT, the dominant motif type for DNRs, was accounted for 86.67% of the total Poaceae species, while AAAT/ATTT for TeNRs was just with the proportion of 40.00%. Furthermore, the microsatellite abundances increased significantly as the motif repeat number decreased, which might be because longer repeats have higher mutation rates and hence are more unstable.

Genic microsatellites, derived from transcripts, have some intrinsic advantages over genomes because of their higher level of transferability to related species, also higher quality and robustness of the amplification product^9^,^27^. We have characterized the distribution of microsatellites in coding sequences across 8 Poaceae species and 3 other plants (Table 2). The results revealed that an overall lower frequency was found in coding sequences when compared to noncoding regions, which might be attribute to negative selection against frameshift mutations in coding regions^28^. It should be noted that 40.26% of the rice gene contained microsatellites and more than half of them (21.46%) were located in the coding region. On the other hand, fewer microsatellites were found in wheat and its two progenitors (*T. urartu* and *A. tauschii*), according for 10.62%, 5.95% and 6.85% of their total gene respectively. Gene Ontology (GO) enrichment analyses of genes containing microsatellites in coding region revealed enrichment in biological regulation (GO: 0065007), pigmentation (GO: 0043473) for Biological Process and binding (GO: 0005488), transcription regulator activity (GO: 0030528) for Molecular Function across these plants. While microsatellites seemed significantly less abundant in genes for catalytic activity (GO: 0003824) in plants (Table S1).

### Frequency of microsatellites in sequenced wheat and its two progenitors

The available 4763.50 Mb (Version: IWGSC2.27, December 2015), 4660.79 Mb and 4147.41 Mb genome sequences of *T. aestivum*, *T. urartu* and *A. tauschii* respectively, were searched for microsatellites with different types of desirable repeat motifs from mono- to hexa-nucleotide (Table 1). A total of 509,321, 456,045 and 422,271 microsatellites (mononucleotide to hexanucleotide) were identified, with an overall frequency of 106.92, 97.85 and 101.82 per Mb or one every 9.52, 10.22 and 9.82 Kb, respectively. Meanwhile, more than 88% of them were perfect repeats, with the number of 454,514, 402,921 and 371,791 for *T. aestivum*, *T. urartu* and *A. tauschii*, respectively.

**Figure 1:**
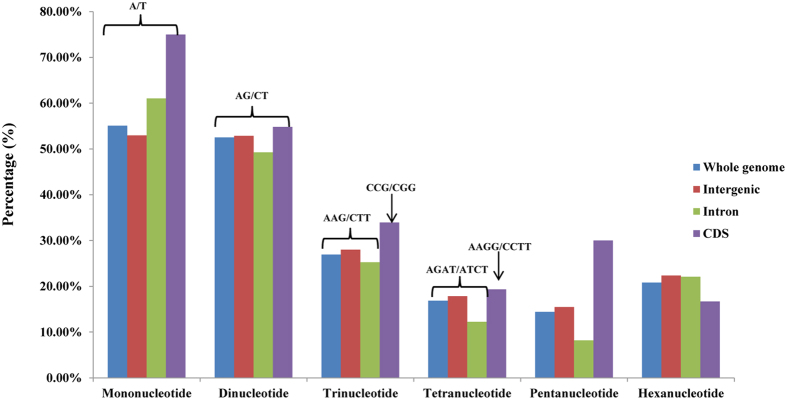
The distribution of the dominant/major motif type of microsatellites in coding and non-coding regions of wheat genome.

Analysis of the distribution of microsatellites across the three subgenomes in wheat revealed that microsatellites were more abundant in the B and D genome chromosomes relative to A (B > D > A) (Table S2). Furthermore, high similarity was observed for several characteristics of microsatellites investigated in the different chromosomes of wheat. MNRs mostly contributed to the proportion of SSRs, and a very small part was contributed by PNRs and HNRs. Among MNRs, more than 90% of the wheat chromosomes were rich in A/T type while G/C was scarce. In the DNRs and TNRs category, the distribution of dominate motif type was perfectly uniform among different wheat chromosomes and the most frequent motif type was AG/CT and AAG/CTT, respectively. While there were more variation for TeNRs, PNRs and HNRs, which have 3 to 5 dominate motif type for them among different chromosomes. For example, for TeNRs the AAAT/ATTT, AATT/AATT, AGAT/ATCT, ACAT/ATGT and ATGC/ATGC were observed more frequently in different wheat chromosomes (Fig. 1). This might be the frequency of TeNRs, PNRs and HNRs was very low in all the wheat chromosomes and their motif-wise distribution was not significant. Although majority characteristics of microsatellites among different chromosomes showed highly similar, the densities of repeats were varied between different chromosomes in wheat. The highest frequency of microsatellites was identified in wheat chromosome 2D (131.03 SSR/Mb) followed by 3B (120.43 SSR/Mb), whereas the lowest frequency was observed in 3 A (82.48 SSR/Mb), with a variation of 1.43-fold (Table S2).

**Figure 2:**
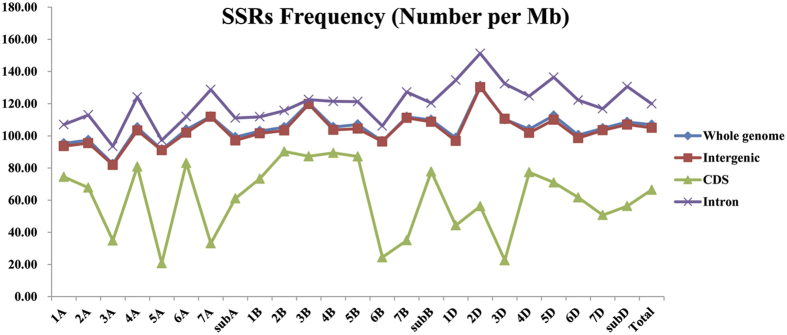
The frequency of microsatellites in coding and non-coding regions among wheat chromosomes.

The distributions of microsatellites in coding and non-coding region were also compared between each wheat chromosomes. Results showed that clear similarity were found for the whole genome and non-coding sequences (including intergenic sequences and intron sequences), but obviously different for the coding sequences. Intron regions showed the highest frequency of microsatellites, whereas coding regions showed the lowest, with a variation of 1.81-fold. Intergenic sequences and whole genome have a similar frequency, with the number of 106.92 and 104.99 per Mb, respectively (Fig. 2). Furthermore, coding sequences have the highest variation (4.35-fold) among different chromosomes in wheat, ranging from 20.74 (5 A) to 90.23 (2B). While there were just 1.59, 1.59 and 1.62-fold change in whole genome, intergenic sequences and intron sequences respectively (Table S2). Excepting for microsatellites frequency, the distributions of different microsatellite motif type were also showed a significant difference between coding and non-coding regions. In all of the wheat chromosomes, TNRs were very frequent in coding sequences, and the most common among them were CCG/CGG (Fig. 1). While MNRs were predominant and AAG/CTT was the most prevalence motif type for TNRs in non-coding sequences (including intergenic sequences and intron sequences). These results were well agreed with the earlier reports in rice^19^, Brachypodium^21^ and switchgrass^24^.

**Figure 3:**
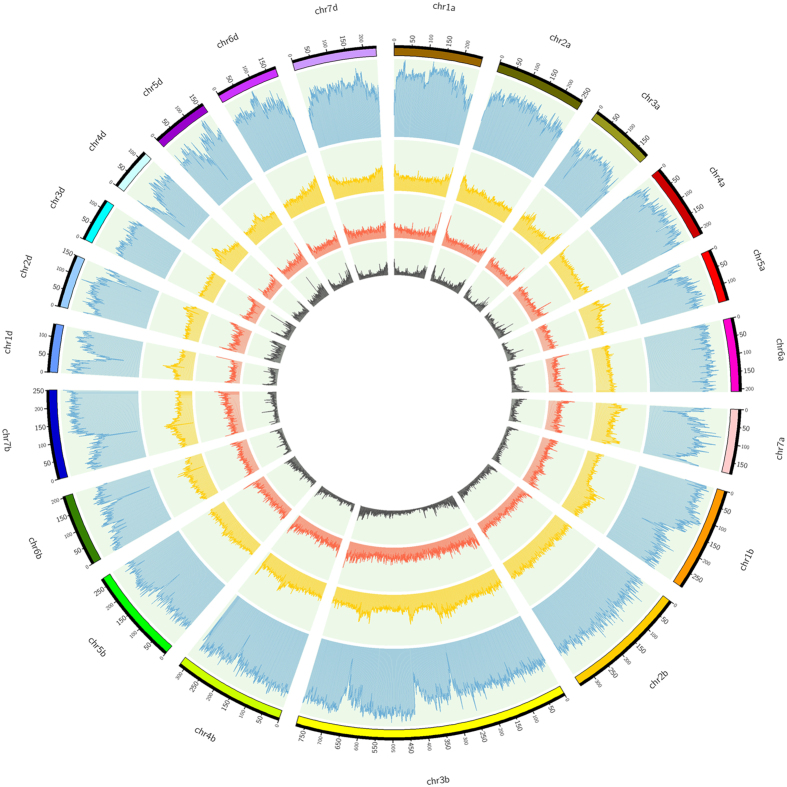
The distribution of microsatellites, genes and TEs in the genome of wheat. From inner to outer: number of genes, number of microsatellites ≥20 bp, number of total microsatellites and number of TEs.

Based on the assembled pseudochromosomes of bread wheat, the genomic distributions of microsatellites and their relation with the annotated genes and TEs were investigated. Results showed that greater physical densities of microsatellites were found in distal chromosomal regions than in the central regions, which was similar to the previous reports in *Gossypium*^23^,^29^,^30^,^31^ and *Brassica* crops^22^. Specifically, the genomic distribution of microsatellites was positively correlated with genes and negatively correlated with TEs (Fig. 3). For wheat, the frequencies of microsatellites in the 1-Mb genomic intervals were significantly positively correlated with genes (r = 0.61) and negatively correlated with TEs (r = −0.50). These results are similar to previous reports that microsatellites are associated with gene sequences in plants^16^,^31^. Furthermore, the flanking sequences (1000 bp) at both sides of each microsatellite were extracted and used to analyze repeat elements with the RepeatMasker Program (RepeatMasker libraries version: rm-20120418). Compared with whole genomic sequences, we observed 11.90% reduction in the class I elements (retroelements) content associated with these flanking sequences, whereas class II elements (DNA transposons) increased 45.03% (Table 3). Gypsy long terminal repeat (LTR) retrotransposons reduced 22.05% and accounted for the greatest proportion of reduction in class I elements, while CMC-EnSpm was the most enriched repeat element type in class II elements (increased 45.99%).

### Development and evaluation of genome-wide SSR markers in wheat and its relatives

The 433,362 perfect microsatellite containing sequences were screened for suitable forward and reverse primer pairs at either side of the flanking genomic sequences. A total of 402,455 microsatellite markers were designed from the genomic sequences of wheat, with successful primer designing potential of 92.87%, which was similar to that documented in foxtail millet^20^ and *Gossypium* species^23^. The physical location of these microsatellites markers was unevenly distributed on 21 chromosomes of *T. aestivum*, with average marker density of 84.49 markers per Mb.

Then, the high-density microsatellite markers were used to investigate the relationships among wheat and its relatives with *in silico* PCR. It was not surprising that the closer relatives will have higher PCR amplification effectivity. As all other species studied here belong to the same genus of *Triticum* (wheat) or the genus of its donor (*Aegilops*), *H. vulgare* showed the lowest PCR amplification effectivity was acceptable. *A. tauschii* was found to be the closest species to *T. aestivum*, followed by *T. urartu*, *T. turgidum* (Table 4). In A-genome lineage species, *T. monococcum* (A^m^ genome) and *T. urartu* (A^u^ genome) was both bearing the A genome, both of which have been implicated as the source of the A genome in polyploid wheat^32^. It has been argued that *T. urartu* and *T. monococcum* are the same species and (or) that the source of the A genome in polyploid wheat is *T. urartu* rather than *T. monococcurn*^32^. Generally, majority of studies supported that *T. urartu* is the donor of the A genome to polyploid wheat^9^,^32^,^33^,^34^. In the present study, *T. monococcum* and *T. urartu* showed significantly different outcomes for markers derived from wheat A subgenome. In *T. urartu* species, nearly half of the markers (47.12%) developed from the wheat A subgenome could amplify prominent PCR products, while it was only 14.92% for *T. monococcum*, with a variation of 3.16-fold change. This result was well agreed with previous report that *T. monococcum* and *T. urartu* were two separately group for the A genome^9^, also confirming that *T. urartu* is the most probable ancestor of the A genome of polyploid wheat^32^,^33^.

The origin of the B genome of polyploid wheat remains controversial and nearly all the Sitopsis genome (S genome) species have been suggested as the donor of the B genomes, including *Aegilops speltoides* (genome S), *A. bicornis* (genome S^b^), *A. longissima* (genome S^l^), *A. searsii* (genome S^s^), and *A. sharonensis* (genome S^sh^)^32^. However, several studies indicated that the B genome of wheat has significantly diverged from all potential extant wild progenitors, although it is closer to *A. speltoides* than to any of the other Sitopsis genomes^35^,^36^. Hence, the majority of evidence seems to suggest that *A. speltoides* is the most likely living relatives of B genomes donor species^9^,^10^. In the present study, two B-genome lineage species (*A. speltoides* and *A. sharonensis*) were investigated to clarify the relationship between them and polyploid wheat. Our results indicated that *T. aestivum* was more closely related to *A. speltoides* for the origin of the B genome, while *A. sharonensis* showed a higher PCR amplification effectivity for markers derived from wheat D subgenome (Table 4). However, only 15.94% of the marker derived from wheat B subgenome could amplify successful in *A. speltoides*, which was significantly lower than that for *T. urartu* (47.12%, 2.96-fold change) and *A. tauschii* (72.34%, 4.54-fold change). Furthermore, there was just slightly higher amplifying rate for markers derived from wheat B subgenome than that for A and D subgenome. This result supported that *A. speltoides* was more likely living relatives of B genome donor species than other speices, but it may not be the direct B donor of *T. aestivum*.

In D-genome lineage species, data obtained in this study also supported the viewpoint that *A. tauschii* was the donor of the D genome for hexaploid wheat^9^,^11^. In addition, it was interesting to find a significantly higher amplifying rate for markers derived from wheat D subgenome than that in A subgenome for its donor, with a variation of 1.54-fold change (Table 4), which was mainly due to the characteristic of microsatellite and the special emerge process of hexaploid wheat. Previous studies showed that microsatellites were subjected to a high rate of single-motif insertion and deletion mutations, through the process of replication slippage^37^,^38^, indicating that microsatellite was in a constant state of change. Compared with wheat D subgenome, A and B subgenome have a longer species differentiation from its progenitor, especially for the selective pressure throughout two ploidisation processes. The later join of D genome to hexaploid wheat might make it remain more ancestral species’ microsatellite characteristics. On the other hand, previous study has showed that little genetic differentiation was found among the D genomes of *T. aestivum* and it appeared to share a single D genome genepool in the evolution of *T. aestivum*^11^. The *A. tauschii* genome sequencing material (AL8/78) has been demonstrated to be one of the closest accessions to wheat D subgenome^39^. Compared with A subgenome donor, the closer relationship combined with bottleneck might be another factor contributed to the higher amplifying rate for markers derived from wheat D subgenome.

**Figure 4:**
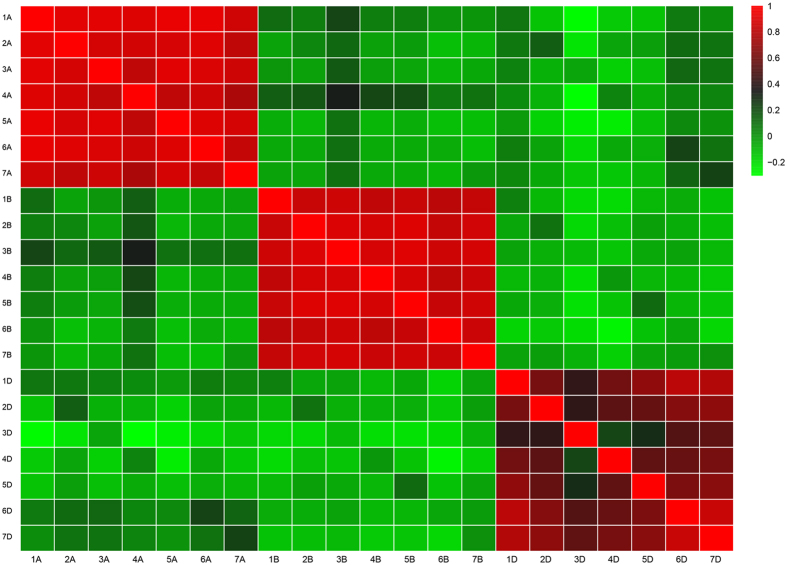
Relationships between chromosomes of wheat revealed by microsatellites. The values are expressed as the total number of microsatellite markers which could have products in the corresponding chromosome and were transferred to log10 scale for clustering.

**Figure 5:**
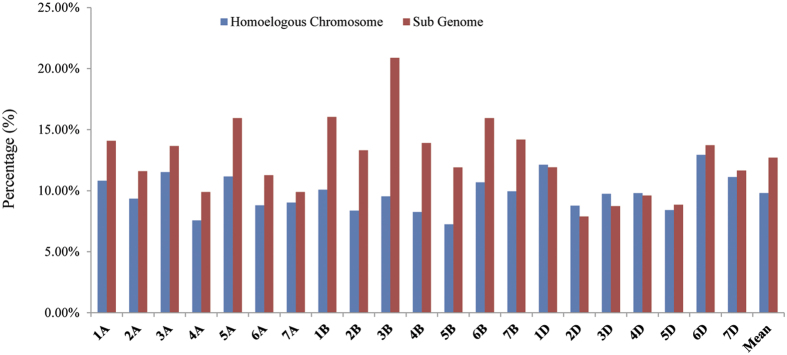
Different average amplification rates between wheat homoeologous chromosomes and its sub genome revealed by newly developed SSR markers. Note: all the developed genome-wide microsatellite markers derived from each chromosome were separately searched against the assembled genomic sequences of wheat with *in silico* PCR. The average amplification rate for homoeologous chromosomes and its sub genome chromosomes were separately calculated for each chromosome. For example, in wheat 1A chromosome, the average amplification rate for homoeologous chromosomes were defined as mean amplification rate in 2A to 7A, while 1B and 1D for its sub genome chromosomes.

In addition, all the developed genome-wide microsatellite markers were also subjected to *in silico* PCR analysis among wheat different chromosomes. Nearly all the microsatellite markers (average success rate 99.88%) could have products in their initial chromosome, while only 10.01% markers could successfully amplify in other chromosomes (Fig. 4). Then, we calculated the average amplification rate for homoeologous chromosomes and its subgenome chromosomes. It was widely accepted that there would be more similar among wheat three homoeologous chromosomes than its subgenome chromosomes. However, a significantly higher PCR amplification effectivity was found in chromosomes at the subgenome than its homoeologous, excepting four chromosomes (1D, 2D, 3D and 4D) (Fig. 5). For example, in wheat 1 A chromosome, an average of 13.19% amplification rate was found in 2A to 7A (subgenome chromosomes), while there were only 9.32% for its homoelogous chromosomes (1B and 1D). As we know, hexaploid wheat was relative new species, indicating that wheat subgenome might have higher progenitor’s characteristics for microsatellites than its new immerge. Furthermore, previous study displayed that the number of the putative translocation events in the wheat D subgenome was about half of those presented in either the A or B subgenome, and majority of the translocations were occurred among chromosomes in the same subgenome^40^. Compared with D subgenome (especially for 1D, 2D, 3D and 4D), the more interchromosomal communications among chromosomes in the same subgenome for A and B might contribute their higher amplification rate in sub genome chromosomes than their homoelogous chromosomes.

**Figure 6:**
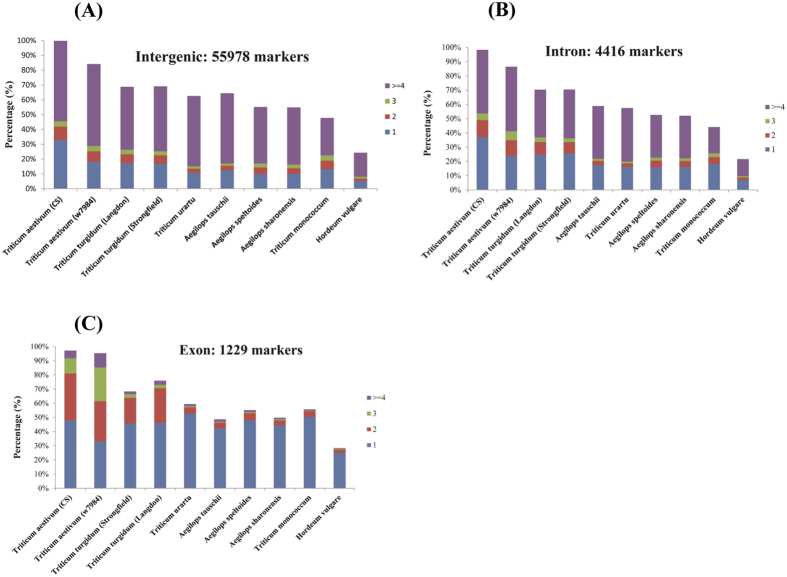
Variation in the numbers of *in silico* PCR products with the newly developed SSR markers in wheat and its relatives. Note: The newly developed SSR markers were evaluated by search against the assembled genomic sequences of wheat and its close relatives with *in silico* PCR. And 1, 2, 3 and ≥4 corresponding to markers generated 1, 2, 3 and ≥4 in silico PCR products in the assembled genomic sequences respectively.

On the other hand, only the longer perfect repeats (SSRs ≥ 20 nucleotides in length) and non-mononucleotides were selected to final genome-wide SSR markers development in wheat for improving their utilization efficiency. Mononucleotides were not considered due to the difficulty of distinguishing bona fide microsatellites from sequencing or assembly error, and because (A/T)n repeats in coding region may be confused with polyadenylation tracks. SSR markers derived from longer perfect repeats (SSRs ≥ 20 nucleotides in length) have demonstrated to show high polymorphic by the experimental data in many organisms such as human^41^ and rice^42^. Finally, a total of 61623 microsatellite markers were designed from the selected genomic sequences of wheat, with a successful rate of 87.36%. Majority of the markers derived from Intergenic region (55978, 90.84%), followed by Intron region (4416, 7.17%) and Exon region (1229, 1.99%) (Fig. 6).

Bread wheat is hexaploid with 21 pairs of chromosomes, being derived from a combination of three diploid donor species via two ploidisation processes. In bread wheat, SSR markers usually amplify multiple fragments from homoeologous DNA sequences, which could complicate or cause errors in the genotype scoring. Therefore, all the newly developed SSR markers (61623) were subjected to *in silico* PCR analysis in the assembled genomic sequences of *Triticum aestivum*. A total of 20169 markers could generated 1 *in silico* PCR products in CS, of which 18216 (90.32%), 1424 (7.06%) and 529 (2.62%) from Intergenic region, Intron region and Exon region respectively (Fig. 6). Furthermore, markers derived from the Exon region (529, 43.43%) displayed the highest amplification rate of unique single allele *in silico*, followed by Intergenic region (18216, 32.54%) and Intron region (1424, 32.25%). While 215 (0.35%), 5964 (9.68%), 2388 (3.88%) and 32394 (52.57%) markers generated 0, 2, 3 and ≥4 in silico PCR products from the survey sequences of CS. Similar results were also found when used these markers search against wheat close relatives (Fig. 6). Genetic and breeding studies demonstrated that microsatellite markers generate one *in silico* PCR product could be especially useful^23^. Therefore, all the 20169 newly developed SSR markers would be used as potential SSR markers in wheat, further used to next polymorphisms evaluation in wheat and its relatives.

To generate microsatellite markers with the potential to direct use, we tested the polymorphisms of 20,666 specific microsatellite makers (generated 1 *in silico* PCR products in CS) in CS and w7984 using the genome sequencing data of w7984 (9.1 Gbp)^43^. A total of 8894 markers generated 1 *in silico* PCR products shared with CS and w7984, with a proportion of 43.07% (8894/20666). To avoid complicated errors in genotyping due to random amplification, all the sequences with 1 *in silico* PCR products in w7984 (8894) were further extracted and used to analyze their microsatellite characteristics (Table 5). For a given primer pair, only the amplicon flanked an SSR with the same basic motif as expected would remain and evaluate their polymorphisms in CS and w7984. Finally, a total of 5478 newly developed SSR markers shared the same microsatellite type in CS and w7984, of which 3267 (59.64%) displayed polymorphisms (Table S2). The high-density SSR marker-based physical maps constructed in this study could be useful for the rapid selection of genome-wide SSR markers that are well distributed over these chromosomes for various genotyping applications (Figure S2).

Furthermore, we also tested the transferability of 20666 newly development microsatellite markers in wheat relatives and barley. To avoid random amplification and multiple fragments, the transferable markers should meet the following criterial: I) the markers should generate 1 *in silico* PCR product; II) the amplicon flanked an SSR with the same basic motif as expected. *In silico* analysis demonstrated that 5836 (28.24%) markers displayed a high level of transferability at any of the seven wheat relatives, of which 4066 (69.67%) markers displayed polymorphisms at least one of the related species test (Table 5). Of the 5836 microsatellite markers, transferability to closely related Triticeae species ranged from 1.53% (316/20666) for *A. sharonensis* to 11.32% (2340/20666) for *T. turgidum* (Langdon) and lower for more distant relatives such as barley (0.18%, 37/20666). Overall, a total of 5836 newly developed genome-wide wheat SSR markers (especially the 4066 polymorphism markers) would also be useful for the closely related Triticeae species (Table S3).

### Validation of SSR markers by PCR amplification

**Figure 7:**
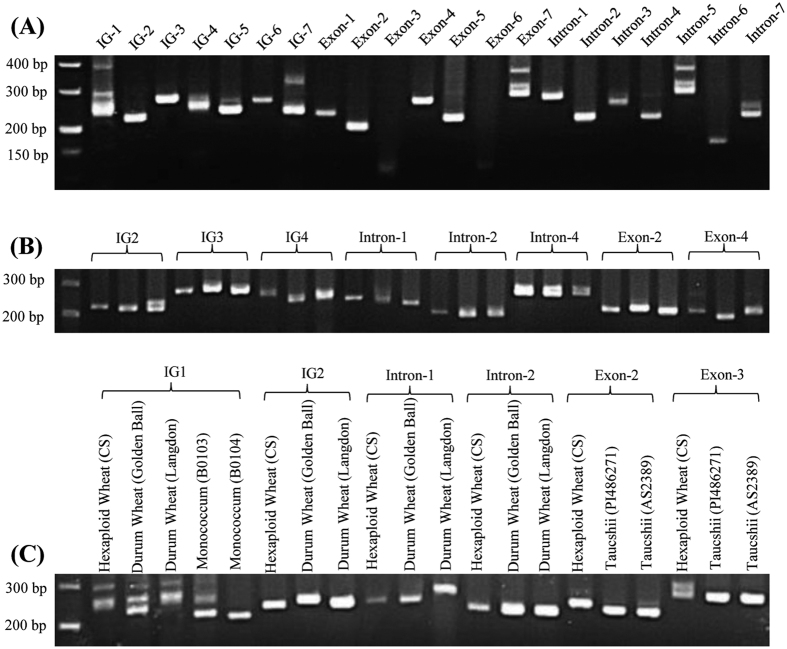
Amplification profiles of newly designed microsatellite marker for wheat and related species by agarose gel electrophoresis. (**A**) 21 microsatellites in ‘Chinese Spring’. (**B**) Eight loci in ‘Chinese Spring’, ‘w7984’ and ‘Opata’ respectively. (**C**) Six loci in wheat and its close relatives.

The validation of newly developed SSR markers in the wheat genome (CS) were performed using 21 randomly selected SSR markers. Nineteen (90.48%) of 21 primer pairs gave clear, successful and reproducible amplification as expected products size (Fig. 7A and Table S4), while 2 markers displayed a weak band at the expected position as a consequence of multiple loci amplification. One of the reasons might be that the ePCR would underestimate the complexity of wheat and there were more multiple loci amplification in real PCR than ePCR. On the other hand, the incomplete wheat genome sequences used in this study might also contribute to underestimate the complexity of wheat. To evaluate polymorphism and molecular diversity potential of developed SSR markers, 8 validated microsatellite markers (3, 3 and 2 markers derived from Intergenic, Intron and Exton region respectively) were amplified in 3 accessions of wheat (CS, w7984 and Opata) (Table S4). All the markers showed polymorphism in these 3 wheat accessions (Fig. 7C). Furthermore, 6 makers which displayed transferable to wheat relatives were validated by PCR amplification (Fig. 7C and Table S4). Thus, high successful amplification rate in wheat and its relatives demonstrated that the newly developed 20666 microsatellite markers for hexaploid wheat genome were a useful resource for wheat genomics and molecular breeding, as well as other Triticeae species.

### Integrated the newly developed markers and other publicly available hexaploid wheat markers into the wheat genome sequence

Recently, SNP markers identified from recent whole-genome shotgun, transcriptome sequencing and genotyping by sequencing (GBS) have been widely used to high-throughput genotyping using DArTSeq technology in wheat such as 90 K SNPs and 400 K SNPs arrays. To enhance the newly developed microsatellite markers as a genomic resource for the wheat genetics and breeding community, we anchored wheat 90 K SNPs, 400 K SNPs and other publicly available microsatellite markers on the same genomic sequence of CS. Finally, a total of 119,576 markers loci were anchored to the genomic sequence of CS, including 119,576 SNP marker loci (12,725 90 K and 106,752 400 K SNP markers) and 99 publicly available microsatellite markers (Table S5). Because the majority of these markers were widely used now or have been anchored to any phenotypic maps, integrating them and the newly development microsatellite markers allowed immediate association newly developed markers to traits targeted by breeders.

## Conclusion

In present study, we conducted a genome-wide analysis of microsatellites in 8 Triticeae species and 9 model plants. The origin, distributions and evolution of microsatellites in *Triticum* species have been characterized and compared. Furthermore, *in silico* PCR of the microsatellite loci was used to analyze the relationship among wheat and its relatives as well as the wheat sub-genome and homoeologous chromosomes, which shed light on the evolution of polyploid wheat from the perspective of microsatellites. Additionally, 20,666 chromosome-specific SSR markers were developed, and their amplification efficiency and polymorphics were investigated in wheat (CS and w7984) as well as other Triticeae species. Among them, 3267 and 4066 markers displayed polymorphisms in wheat different materials (CS and w7984) and its close relatives, respectively. Finally, the newly developed SSR markers were further integrated with the publicly available wheat markers to dense the wheat genetic map. Our study not only provided the rich resource for SSR markers development for wheat and Triticeae species, but also provided the important information on the evolution of polyploid wheat.

## Materials and Methods

### Sources of genome sequences

The whole genome sequences of *Triticum aestivum* (CS), *T. monococcum*, *Aegilops sharonensis*, *A. speltoides*, *T. turgidum* (Strongfield) and *T. turgidum* (Langdon) were retrieved from URGI (https://urgi.versailles.inra.fr/) and the assembly sequences of *T. aestivum* (w7984) were obtained from dx.doi.org/10.5447/IPK/2014/14. The remaining genomic sequences of 12 species were downloaded from Gramene database (ftp://ftp.gramene.org/).

### Identification of microsatellites and primer design

Genome sequences were searched for microsatellites using the default parameter of MISA identification tool^27^. The search criteria were: six repeat units for dinucleotide repeats (DNRs), five repeat units for trinucleotide repeats (TNRs), tetranucleotide repeats (TeNRs), pentanucleotide repeats (PNRs) and hexanucleotide repeats (HNRs). Compound microsatellites were defined as ≥2 repeats interrupted by ≤100 bp.

The forward and reverse primers flanking the identified microsatellite repeat motifs were designed in batches using the primer3_core program. Two perl scripts, p3_in.pl and p3_out.pl, serve as the interface modules for the programmer-to-programmer data interchange between MISA and the primer modeling software Primer 3.0^44^. The major parameters for primer design were as follows: 18–27 bp in primer length, 57–63 °C in melting temperature, 30–70% in GC content and 100–300 bp in product size.

### Statistical analysis and functional annotation

The repetitive elements were detected using RepeatMasker Program against RepBase (Version: rm-20120418) database with defaulting parameters. TE families were classified as previously described^45^. Each chromosome was divided into 1-Mb for statistical analysis of microsatellites, genes and TEs for the represent practical frequencies. The Circos-0.67-7 software was used to visualize the frequencies of microsatellites, genes and TEs in wheat 21 chromosomes^46^. Statistical analyses were performed using SPSS Statistics 17.0 (SPSS Japan, Inc., Tokyo, Japan). Functional annotation of genes containing microsatellites was performed by using Gene Ontology Tools.

### *In silico* evaluation of genome-wide SSR markers in wheat and its relatives

In order to improve the utilization efficiency of newly development SSR markers, only the markers with longer perfect repeats (SSRs ≥ 20 nucleotides in length) and non-mononucleotides were selected to *in silico* analysis of their polymorphisms in wheat and its relative species. The software (e-PCR-2.3.12) was used for *in silico* PCR analysis with the following parameters: 7 word size, 0 discontinuous word, 50 bp margin, 2 mismatch, 1 gap, and 100 −300 bp product size^47^. Only one genome was used at once. The paired primer regarded as a putative polymorphic primer should meet the following criterial: I) generated 1 *in silico* PCR product at any test genome; II) the amplicon flanked an SSR with the same basic motif as expected; III) the length of PCR product variation derived from the alteration of microsatellites motif. Hierarchical clustering was visualized on heatmaps in R using the gplots package (http://www.R-project.org), specifying average linkage and Pearson’s correlation distance metric.

### Validation of SSR markers by PCR amplification

A total of 21 SSR primer pairs were synthesized to test for PCR amplification in wheat and its close relatives. Genomic DNA of these selected materials was isolated from young leaves by a standard procedure^48^. PCR amplification reactions were performed in 20-ul volume that contained 1 ul template DNA (100 ng), 0.5 ul of each primer (10 uM), 1.6 ul dNTP (2.5 mM each), 1.6 ul MgCl_2_ (25 mM), 0.2 ul *Taq* DNA polymerase (5 U/ul), and 2ul *Taq* buffer (10×). DNA amplification was conducted by the ‘touchdown’ method with two stages: stage I) initial denaturation at 94 °C for 5 min followed by six cycles of denaturation at 94 °C for 30 s, annealing at 63 °C for 45 s with a 1 °C decrease in each subsequent cycle and extension at 72 °C for 1 min; stage II) 26 cycles of 30 s at 94 °C, 45 s at 57 °C and 1 min at 72 °C and a final extension at 72 °C for 10 min. The PCR products were separated on 3% agarose gels and were visualized by ethidium bromide staining.

### Integrated the newly developed polymorphic SSR markers with 90 K SNPs, 400 K SNPs and other publicly available SSR markers

A total of 1705 SSR markers were successfully downloaded from GrainGenes database (http://wheat.pw.usda.gov/GG3/) and subjected to search against the whole genome sequences of wheat (CS) using an *in silico* PCR strategy. The software (e-PCR-2.3.12) was used for in silico PCR analysis with the following parameters: 7 word size, 0 discontinuous word, 50 bp margin, 2 mismatch, 1 gap, and 100–300 bp product size. Only the markers mapped to unique locations in the reference wheat survey genome (CS) were remained.

In addition, 458,919 SNPs markers were downloaded from GrainGenes database (http://wheat.pw.usda.gov/GG3/), of which 37,853 markers for wheat 90 K SNPs and 421,066 for 400 K SNPs. These SNP markers were mapped against wheat survey sequence (CS) using BLAT (v.34) software^49^. Criteria for assigning chromosomal locations of SNP markers as described by Mayer *et al*. (2014) with some modifications^14^: I) 95% identify, 95% coverage and gap rate less than 2%; II) Only the markers mapped to unique location in the reference wheat survey genome (CS) were remained.

## Additional Information

**How to cite this article**: Deng, P. *et al*. Genome-wide characterization of microsatellites in Triticeae species: abundance, distribution and evolution. *Sci. Rep.* **6**, 32224; doi: 10.1038/srep32224 (2016).

## Supplementary Material

Supplementary Information

Supplementary Table S1

Supplementary Table S2

Supplementary Table S3

Supplementary Table S4

Supplementary Table S5

## Figures and Tables

**Table 1:** The number and frequency of microsatellites in the whole genomes of 14 sequenced Poaceae species and three other plants.

Species	Whole genome						Microsatellite Type (%)			
	Genome Size (Mb)	GC Content (%)	SSRs Number	SSRs Frequency (Number per Mb)	MNRs	DNRs	TNRs	TeNRs	PNRs	HNRs
*Brachypodium distachyon*	271.92	46.21	51981	191.16	**58.95**	18.12	20.48	1.90	0.38	0.16
*Oryza sativa*	373.25	43.55	135501	363.03	**47.86**	27.57	22.04	1.90	0.45	0.19
*Setaria italica*	405.74	45.6	61931	152.64	**54.24**	21.11	21.83	2.13	0.44	0.26
*Sorghum bicolor*	738.54	41.49	129564	175.43	**43.15**	29.44	21.98	4.14	0.73	0.56
*Triticum monococcum*	1208.99	47.12	207430	171.57	**48.79**	32.24	16.54	2.07	0.24	0.11
*Aegilops sharonensis*	1603.87	46.21	216638	135.07	**44.02**	33.38	19.94	2.22	0.27	0.18
*Aegilops speltoides*	1760.38	45.97	301460	171.25	**52.54**	29.04	16.12	1.93	0.24	0.12
*Phyllostachys heterocycla*	2051.72	39.35	302615	147.49	**50.20**	37.13	9.81	1.94	0.42	0.51
*Zea mays*	2066.43	46.6	198939	96.27	**43.18**	32.77	21.53	1.67	0.54	0.31
*Triticum turgidum* (Strongfield)	3119.31	46.16	451421	144.72	**47.25**	33.98	16.47	1.95	0.22	0.14
*Triticum turgidum* (Langdon)	3139.70	46.76	472611	150.53	**47.58**	33.09	17.07	1.91	0.22	0.13
*Hordeum vulgare*	1868.65	43.19	289981	155.18	**45.47**	32.12	20.16	1.85	0.27	0.14
*Triticum aestivum* (Version 2, 2014 May)	10138.70	44.63	1064878	105.03	28.97	**47.24**	21.75	1.62	0.20	0.21
*Triticum urartu*	4660.79	38.2	456045	97.85	32.32	**43.75**	21.77	1.59	0.15	0.41
*Aegilops tauschii*	4147.41	37.75	422271	101.82	36.49	**39.83**	21.84	1.45	0.17	0.22
*Arabidopsis thaliana*	119.67	36	50092	418.59	**69.56**	18.74	11.17	0.34	0.08	0.11
*Populus trichocarpa*	417.14	32.57	278606	667.90	**69.83**	19.49	9.02	1.14	0.28	0.24
*Glycine max*	973.34	34.1	405325	416.43	**64.56**	24.64	9.48	0.98	0.29	0.07

**Table 2:** The distribution of microsatellites in coding sequences across 9 grass species and 3 other plants.

Species	Gene Number		Gene Containing SSR (Number and Percent)		Sequence Size (Mb)		SSRs Number		SSRs Frequency (Number per Mb)	
	Gene	CDS	Gene	CDS	Gene	CDS	Gene	CDS	Gene	CDS
*Triticum aestivum*	102503	100344	10884 (10.62%)	4931 (4.91%)	125.79	88.50	12972	5508	103.12	62.23
*Triticum urartu*	34977	33483	2081 (5.95%)	2035 (6.08%)	37.72	36.50	2426	2378	64.32	65.16
*Aegilops tauschii*	33995	33928	2328 (6.85%)	2328 (6.86%)	40.87	40.87	2640	2640	64.59	64.60
*Hordeum vulgare*	62584	62315	13005 (20.78%)	3787 (6.08%)	116.95	65.91	16890	4291	144.42	65.10
*Brachypodium distachyon*	31029	31029	5369 (17.3%)	3717 (11.98%)	49.14	39.73	6492	4443	132.13	111.83
*Oryza sativa*	42132	40745	16961 (40.26%)	8744 (21.46%)	66.78	45.52	26282	12262	393.56	269.37
*Setaria italica*	40599	40599	6748 (16.62%)	4342 (10.69%)	57.96	46.06	8132	5061	140.31	109.88
*Sorghum bicolor*	36338	36338	7825 (21.53%)	5435 (14.96%)	49.07	42.18	10460	6920	213.17	164.04
*Zea mays*	63235	63235	14667 (23.19%)	6673 (10.55%)	97.45	63.28	19635	8221	201.49	129.92
*Arabidopsis thaliana*	40223	35386	9667 (24.03%)	2880 (8.14%)	64.25	43.55	12765	3302	198.68	75.83
*Glycine max*	73319	73319	23119 (31.53%)	5723 (7.81%)	132.77	89.75	32869	6846	247.56	76.28
*Populus trichocarpa*	45778	45778	8745 (19.1%)	2825 (6.17%)	64.49	51.55	11246	3282	174.38	63.67

**Table 3:** Comparative analysis of repeat elements in the flanking sequences (1000 bp) at both sides of each microsatellite and wheat whole genome sequences.

Repeat Class/Family	SSR Flanking Sequences		Total Genome	
	Number of element	Percentage (%)	Number of element	Percentage (%)
**Class I (retrotransposons)**	491245	**59.76**	2488203	**71.65**
LTR	482495	58.69	2446227	70.44
Copia	155464	18.91	673974	19.41
Gypsy	327031	39.78	1772253	51.04
LINE	7973	0.97	39121	1.13
SINE	777	0.09	2855	0.08
**Class II (DNA transposons)**	**127818**	**15.55**	372284	**10.72**
CMC-EnSpm	91908	11.06	263134	7.58
hAT-Tag1	537	0.06	2620	0.08
PIF-Harbinger	7060	0.85	29445	0.85
TcMar-Stowaway	19859	2.39	54371	1.57
**Other repeats**			22714	0.65
low complexity	73548	8.85	29534	0.85
Simple repeat	136250	16.40	368430	10.61
Satelltie	1853	0.22	213477	6.15
Small RNA	118	0.01	650	0.02

**Table 4:** Analysis of the relationship among wheat and its relatives utilizing microsatellites.

Chromosome	Number of total markers	Percentage of markers with PCR products *in silico*						
		*Triticum monococcum*	*Triticum urartu*	*Aegilops speltoides*	*Aegilops sharonensis*	*Aegilops tauschii*	*Triticum turgidum*	*Hordeum vulgare*
1A	17990	15.03%	**49.71%**	10.38%	12.51%	21.27%	**41.92%**	1.56%
2A	19687	15.82%	**47.01%**	9.22%	11.33%	18.99%	**44.44%**	1.44%
3A	11829	15.10%	**48.21%**	10.26%	12.43%	21.55%	**44.46%**	1.94%
4A	17977	12.20%	**39.78%**	9.09%	10.93%	16.90%	**45.75%**	1.47%
5A	10694	14.86%	**53.23%**	9.92%	12.56%	22.88%	**44.70%**	1.88%
6A	16831	17.26%	**46.14%**	9.16%	11.11%	18.58%	**42.75%**	1.50%
7A	14346	14.15%	**45.74%**	8.39%	10.29%	17.09%	**45.34%**	1.40%
1B	22504	5.44%	18.69%	**16.35%**	13.57%	20.52%	**39.49%**	1.69%
2B	28080	5.09%	16.10%	**16.02%**	12.30%	17.57%	**41.43%**	1.58%
3B	68773	5.80%	24.47%	**16.94%**	15.00%	24.75%	**32.05%**	1.49%
4B	24604	4.88%	16.55%	**15.28%**	12.31%	18.30%	**38.97%**	1.65%
5B	22897	5.26%	14.56%	**15.84%**	12.25%	16.33%	**45.82%**	1.69%
6B	15076	5.08%	18.89%	**16.05%**	13.60%	20.68%	**42.78%**	1.75%
7B	19348	4.99%	17.71%	**15.09%**	12.35%	19.75%	**39.93%**	1.93%
1D	10828	7.89%	21.08%	12.58%	17.18%	**70.57%**	16.35%	2.33%
2D	14320	6.85%	15.27%	10.29%	14.68%	**69.70%**	13.37%	2.14%
3D	10121	6.38%	15.54%	11.11%	14.98%	**69.30%**	13.59%	2.05%
4D	9841	7.96%	17.96%	11.83%	17.95%	**73.49%**	14.65%	2.16%
5D	14416	7.65%	16.90%	11.27%	16.27%	**72.61%**	14.28%	2.15%
6D	13849	8.63%	24.15%	14.33%	19.63%	**76.16%**	17.48%	2.53%
7D	18444	8.09%	20.67%	12.79%	17.73%	**74.53%**	16.17%	2.42%

**Table 5:** The number of newly developed SSR markers with potential to directly use in wheat and its close relatives.

Species	Extron Region		Intron Region		Intergenic Region		Whole genome	
	Unique markers number	Number of Polymorphism	Unique markers number	Number of Polymorphism	Unique markers number	Number of Polymorphism	Unique markers number	Number of Polymorphism
W7984	166	60	460	234	4852	2973	5478	3267
*Triticum turgidum* (Langdon)	151	49	307	136	1882	977	2340	1162
*Triticum turgidum* (Strongfield)	146	55	289	124	1814	939	2249	1118
*Aegilops tauschii*	89	57	147	93	1223	899	1459	1049
*Triticum urartu*	75	42	116	86	803	668	994	796
*Triticum monococcum*	64	45	57	46	336	259	457	350
*Aegilops speltoides*	57	44	70	58	213	179	340	281
*Aegilops sharonensis*	49	40	50	45	217	185	316	270
*Hordeum vulgare*	16	14	9	8	12	11	37	33
Total CS markers							5836	4066
