# Supplementary material for: Genome-wide characterization of microsatellites in Triticeae species: abundance, distribution and evolution
Source: Sci Rep. 2016 Aug 26;6:32224. doi: 10.1038/srep32224 (PMC4999822; doi:10.1038/srep32224)
Supplement: Supplementary Information [file srep32224-s1.doc]

**Supplementary Information for:**

**Genome-wide characterization of microsatellites in Triticeae species: abundance, distribution and evolution**

Pingchuan Deng^1, +^, Meng Wang^1, +^, Kewei Feng^1^, Licao Cui^1^, Wei Tong^1^, Weining Song^1, 2^*, Xiaojun Nie^1^, *

1 State Key Laboratory of Crop Stress Biology in Arid Areas, College of Agronomy and Yangling Branch of China Wheat Improvement Center, Northwest A&F University, Yangling, Shaanxi 712100, China

2 Australia-China Joint Research Centre for Abiotic and Biotic Stress Management in Agriculture, Horticulture and Forestry, Yangling, Shaanxi 712100, China

*Corresponding author:

Weining Song: sweining2002@yahoo.com

Xiaojun Nie: small@nwsuaf.edu.cn

Address: College of Agronomy, Northwest A&F University, Yangling 712100, Shaanxi, China;

Tel: (+86) 29 87082984; Fax: (+86) 29 87080191

^+^ These authors contributed equally to this work


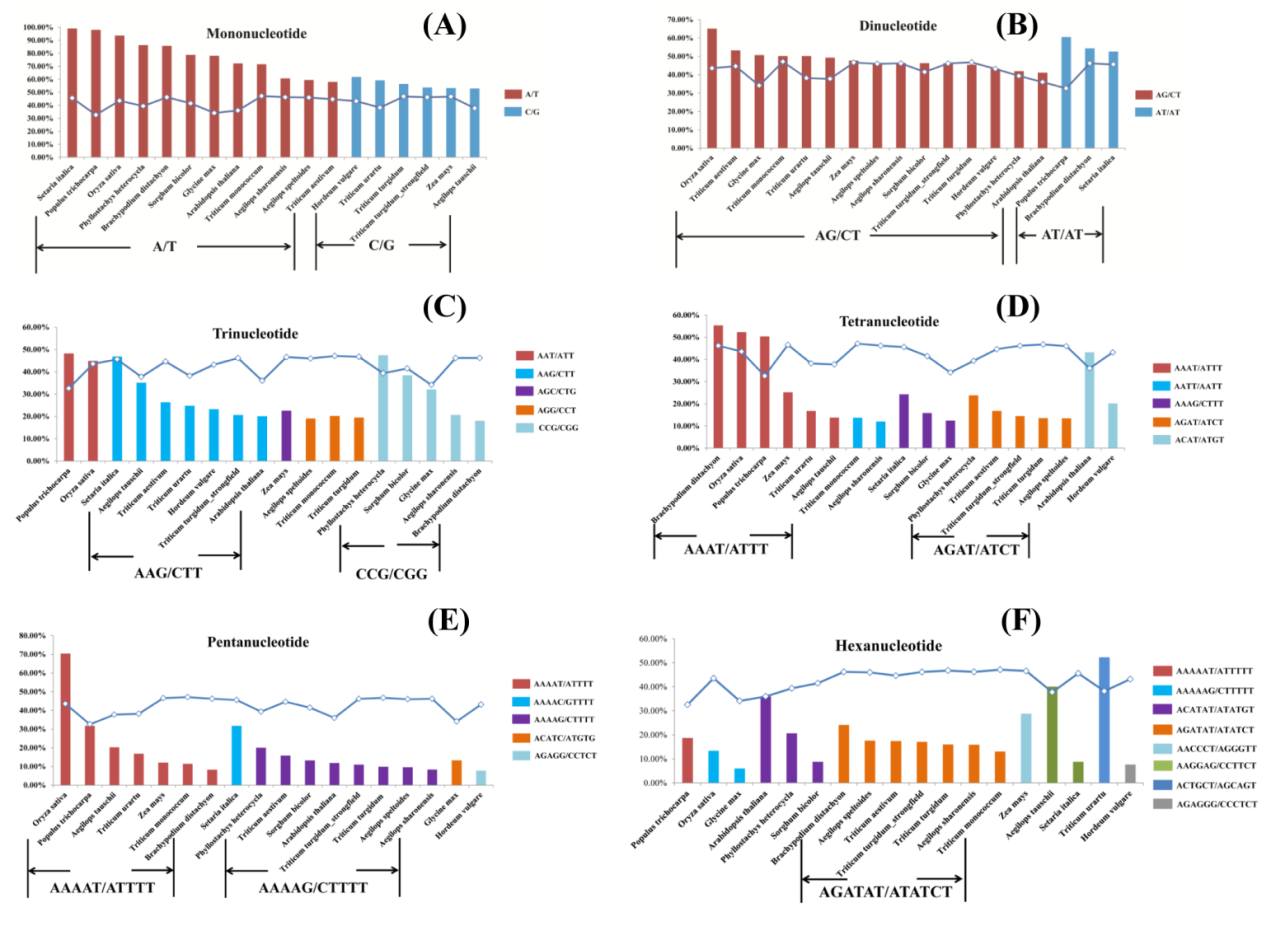


Figure S1. The distributions with respect to the dominant/major motif type of microsatellites in 15 sequenced Poaceae species and three other plants


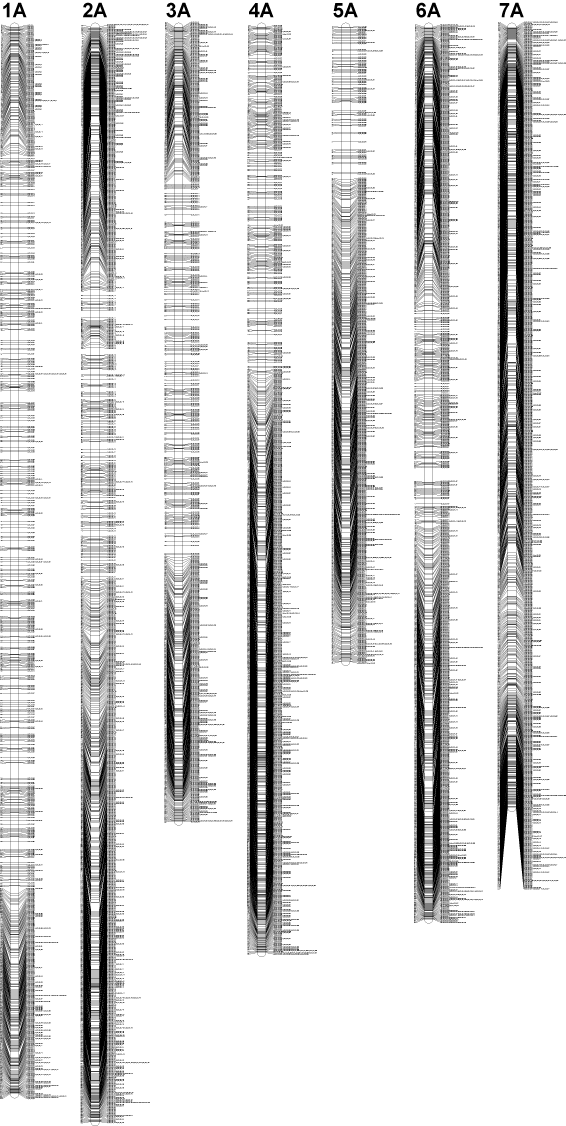


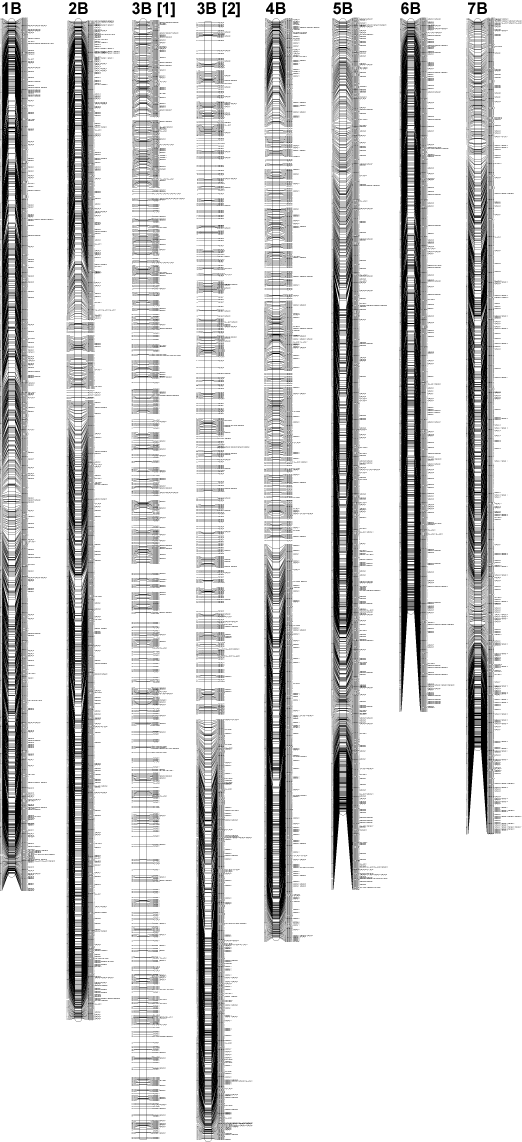


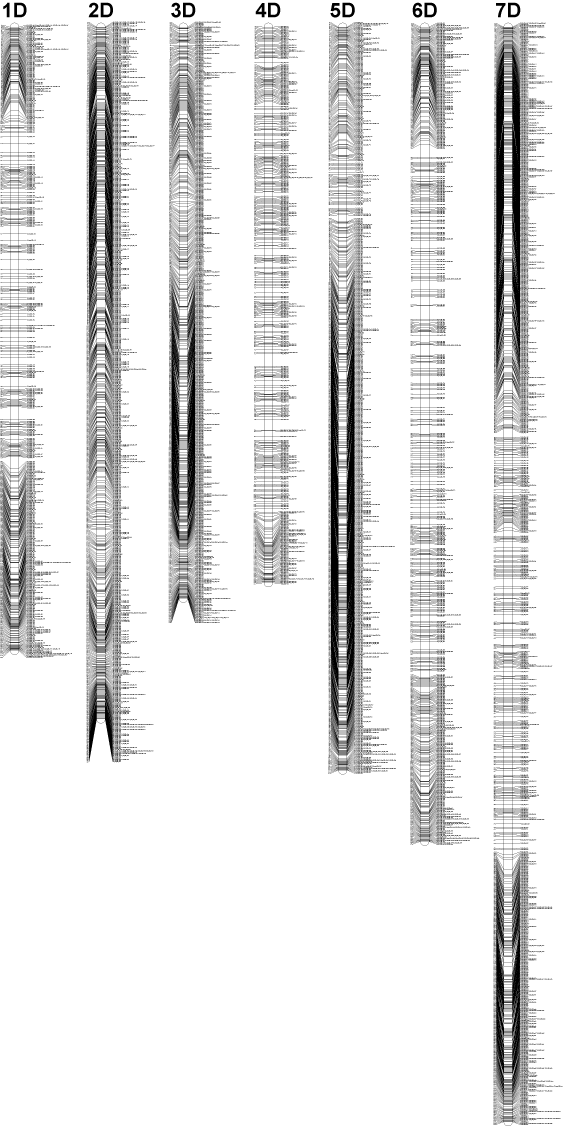
Figure S2. The physical localization of 20,666 genomic microsatellite markers on the 21 chromosomes of wheat (CS)

Table S1. Gene Ontology (GO) enrichment analyses of genes containing microsatellite in 9 grass species and 3 other plants. Note: Number in the table corresponding to Expected and Obeserved value respectively.

Table S2. The number and frequency of microsatellites in coding and non-coding region among each wheat chromosomes.

Table S3. The detail lists of newly developed SSR markers in this study. Note: IGR, Intron and Exon in Marker ID corresponding to marker derived from Intergenic region, Intron region and Exon region in wheat genome (CS); Genome Loci corresponding to chromosome or scaffold loci, product start, product end, motif type and number, polymorphisms or not (yes means polymorphisms between CS and test material and no means non-polymorphic). For example, ‘TGAC_WGS_monococcum_v1_contig_76218:1584-1728:145:(GGAC)5:no’ means this marker could successfully amply in *Triticum monococcum* genome and the product located on contig_76218 (TGAC_WGS_monococcum_v1) from 1584 bp to 1728 bp. The PCR product size was 145 bp and motif type was GGAC with 5 copy number. This marker showed non-polymorphic between CS and test monococcum material.

Table S4. Information of primers used for PCR validation.

Table S5. The detail list of publicly available wheat markers anchored in wheat genome (CS)
